# RDT performance through high-throughput bead-based antigen detection during malaria school survey in Senegal

**DOI:** 10.3389/fpara.2025.1598280

**Published:** 2025-05-29

**Authors:** Mamadou Alpha Diallo, Ibrahima M. Ndiaye, Djiby Sow, Mame Cheikh Seck, Khadim Diongue, Mariama Touré, Katerine E. Battle, Bassirou Ngom, Mouhamad Sy, Amy Gaye, Yaye Dié Ndiaye, Mamane Nassirou Garba, Aida Sadikh Badiane, Aita Sene, Medoune Ndiop, Jules François Gomis, Sarah K. Volkman, Doudou Sene, Bronwyn L. MacInnis, Ibrahima Diallo, Mouhamadou Ndiaye, Dyann F. Wirth, Daouda Ndiaye

**Affiliations:** ^1^ International Research and Training Center in Applied Genomic and Health Surveillance (CIGASS), Cheikh Anta Diop University, Dakar, Senegal; ^2^ Institute for Disease Modeling, Gates Foundation, Seattle, WA, United States; ^3^ National Malaria Control Program (NMCP), Dakar, Senegal; ^4^ Department of Immunology and Infectious Diseases, Harvard T. H. Chan School of Public Health, Boston, MA, United States; ^5^ Infectious Disease and Microbiome Program, The Broad Institute, Cambridge, MA, United States

**Keywords:** malaria surveillance, rapid diagnostic tests (RDTs), high-throughput bead-based antigen detection, school-based surveys, Senegal

## Abstract

**Background:**

Rapid Diagnostic Tests (RDTs) remain the frontline tool for malaria diagnosis, but their performance in detecting low-density infections is variable and poorly characterized at the population level.

**Objective:**

This study aimed to evaluate the diagnostic performance of HRP2-based RDTs by integrating high-throughput bead-based HRP2 quantification into school-based malaria surveys.

**Methods:**

A cross-sectional study was conducted in three Senegalese districts (Diourbel, Tambacounda, and Kédougou), enrolling 3,748 school-aged children. All participants were tested using RDTs, and dried blood spots were analyzed with a multiplex bead-based HRP2 assay. A Gaussian mixture model was used to classify HRP2 positivity, and logistic regression assessed the relationship between HRP2 concentration and RDT outcome.

**Results:**

The overall RDT positivity rate was 7.2%, with marked heterogeneity across districts (Diourbel: 3.0%, Kédougou: 15.9%, Tambacounda: 7.6%). HRP2 concentration was the strongest predictor of RDT positivity (aOR: 14.55 per log_10_ increase, 95% CI: 11.14–19.00). RDT limits of detection (LOD_95_) varied significantly: 3.9 ng/mL in Tambacounda, 121.2 ng/mL in Kédougou, and 204.3 ng/mL in Diourbel.

**Conclusion:**

RDTs remain a useful surveillance tool, particularly in moderate- to high-transmission settings. However, reduced sensitivity at lower antigen concentrations in hypo-endemic areas highlights the value of complementary high-sensitivity assays for elimination-focused strategies. Future research should explore the application of these integrated diagnostic approaches in regions without seasonal malaria chemoprophylaxis intervention.

## Introduction

According to the *World Malaria Report 2023* by the World Health Organization, there were an estimated 263 million malaria cases globally in 2023, marking an increase of 11 million cases from the previous year. The number of malaria-related deaths remained steady at approximately 597,000. Sub-Saharan Africa continues to bear the highest burden, accounting for about 94% of global cases and 95% of deaths. Children under five years old are particularly vulnerable, representing around 74% of all malaria deaths in 2023. Senegal reported 1,199,388 malaria cases and 3,070 deaths in 2023 (WHO, 2024). Vulnerable populations, particularly young children and pregnant women, remain at higher risk for severe malaria complications and death (Bhatt et al., 2015).

National Malaria Control Programs (NMCPs) in countries like Senegal have implemented several preventive strategies, including intermittent preventive treatment and seasonal malaria chemoprevention (SMC). In Senegal, SMC has been a cornerstone intervention aimed at reducing malaria cases by up to 75% in children aged 3–59 months, and its coverage was later extended to children up to 10 years of age (Ndiaye, 2016).

While these control interventions have led to a substantial decline in the malaria burden since 2000, continued surveillance is essential for assessing transmission’s spatial and temporal dynamics—particularly in asymptomatic populations, where individuals carry the parasite without showing symptoms (Bousema et al., 2014)​. This silent reservoir of infection, often detectable only through sensitive molecular techniques, plays a key role in sustaining transmission and can undermine malaria elimination efforts​. In high-transmission regions, asymptomatic *Plasmodium falciparum* parasitemia remains prevalent, and evidence shows that up to 25% of individuals in sub-Saharan Africa harbor submicroscopic parasites capable of sustaining transmission (Nankabirwa et al., 2014). These asymptomatic carriers, who do not seek treatment, contribute to ongoing malaria transmission, making it more challenging to achieve elimination targets. Furthermore, school-aged children (5–15 years old) often harbor these subclinical infections, positioning them as a significant yet under-recognized reservoir for malaria transmission (Brooker et al., 2009).

Molecular diagnostic techniques, while highly sensitive for detecting asymptomatic infections, are impractical for large-scale prevalence studies due to their complexity and resource requirements. Microscopy, although long considered the gold standard, is time-consuming and requires significant expertise, which makes it less suitable for field epidemiology involving thousands of samples. Though user-friendly and deployable in the field, RDTs have relatively limited sensitivity for low-density infections. To address these limitations, a high-throughput bead-based antigen detection method (a one-step multiplex assay) was integrated as a complementary tool to RDTs​. This multiplex immunoassay is a powerful method for detecting malaria antigens and can improve infection identification in large-scale surveillance efforts (Rogier et al., 2019).

In Africa, routine malaria data predominantly come from the health management information system (HMIS) and from large household surveys such as the Malaria Indicator Survey (MIS) and the Demographic and Health Survey (DHS) (Pison et al., 2018). However, HMIS data include only cases reported at health facilities, and national household surveys—conducted every 4–5 years—have a limited scope, focusing solely on children under 5 (Programme National de Lutte contre le Paludisme (PNLP), 2015). Inconsistent funding and weaknesses in health systems further hinder timely, reliable malaria data collection​. Given this context, there is growing interest in utilizing school surveys to complement household surveys for national malaria surveillance (Snow, 2002). Schools are well-organized and easily accessible, offering the opportunity to collect malariometric and intervention coverage data from a large number of children in a short period and at lower cost compared to household surveys​​. The value of school survey data in planning targeted interventions has been demonstrated in other African countries (Brooker et al., 2009). School-based malaria surveys provide an efficient, cost-effective method for assessing malaria prevalence and transmission dynamics in communities​​. Integrating data from school surveys with health facility incidence data can yield a more comprehensive understanding of malaria transmission patterns across different regions (Stevenson et al., 2016)​. Indeed, school-based surveys have proven to be effective in identifying infection hotspots and areas of heterogeneous transmission that might require additional interventions​ (Brooker et al., 2009; Gitonga et al., 2010).

It has been suggested that national household surveys such as the DHS or MIS could rely solely on RDTs to estimate malaria prevalence. In this context, bead-based Histidine-Rich Protein 2 (HRP2) assays have been used as an external validation tool for RDT results in field surveys involving samples from various African regions, including Senegal​​ (Plucinski et al., 2017a; Martiáñez-Vendrell et al., 2020). Bead-based assays have the ability to detect multiple antigens simultaneously and are well-suited for high-throughput screening of large sample sets, highlighting their potential utility in malaria surveillance (Jang et al., 2022). Therefore, this study aimed to integrate this advanced immunological tool with traditional RDT methods to assess their combined effectiveness in determining community-wide infection rates through school-based surveys. It was hypothesized that integrating bead-based antigen detection with RDTs would demonstrate that RDTs capture the majority of malaria infections even at low parasite densities, thereby validating their use in community surveillance.

## Methods

### Study area

**Figure 1:**
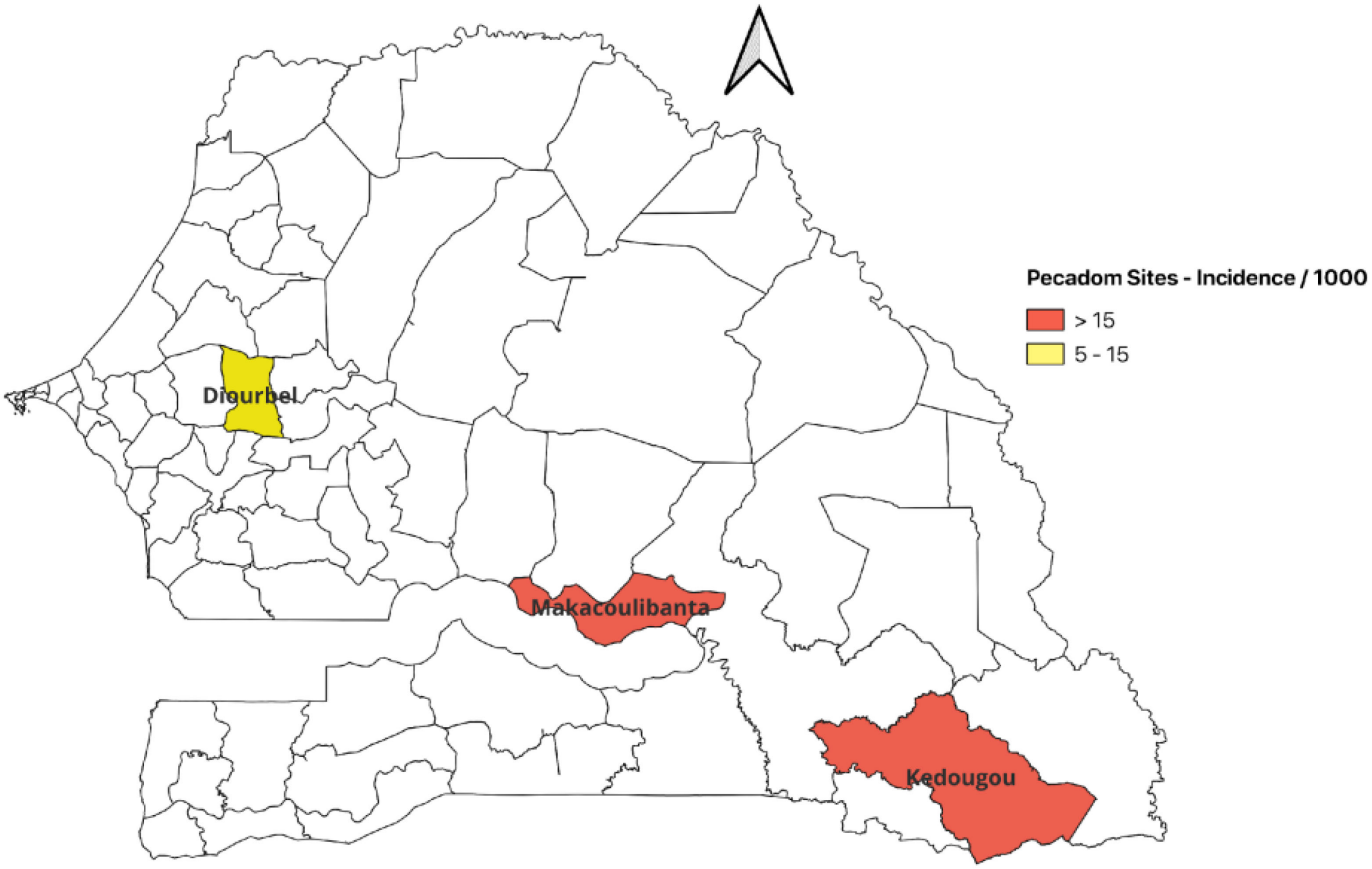
Map of Senegal highlighting the three study districts. Districts in red labeled Pecadom (prise en charge à domicile) have a community health worker program.

This cross-sectional study was carried out in November 2021 in three districts of Senegal: Kédougou, Tambacounda, and Diourbel (**Figure 1**).

Kédougou: Kédougou health district is situated in Kédougou Region in the far southeast of Senegal. It spans an area of 9,984 km² and had a population of 154,471 in 2021, yielding a population density of about 11 inhabitants per km². The district experiences two main seasons: a rainy season from June to October and a dry season from November to May. In 2020, Kédougou received approximately 1,426 mm of total rainfall (regional average ~1,162 mm).

Tambacounda: Maka Coulibantang health district is located in Tambacounda Region in southern Senegal, approximately 418 km from Dakar. The climate is tropical Sudanian with alternating rainy and dry seasons. Average annual rainfall is around 1,000 mm. (Population data for this district were not explicitly recorded in this survey; however, the region has a relatively sparse population compared to Diourbel.)

Diourbel: Diourbel health district lies 145 km east of Dakar, covering an area of 1,175 km². The population of the Diourbel health district is estimated at 354,793 (2021), with a population density of about 302 inhabitants per km². The climate is tropical semi-arid, with a long dry season (November to May) and a shorter rainy season (late June to early October). Annual rainfall is approximately 610 mm. The Diourbel district is in the Baol region, known for the substantial presence of *Mouride* Muslims. It is characterized by socio-cultural and religious factors that can either facilitate or limit health interventions. Diourbel has numerous daaras (Quranic schools), which are traditional boarding schools for boys.

### Study population

The study population consisted of primary school-age children enrolled in selected conventional (government-run) and Quranic (daaras) schools in the three districts. The selection of schools was done in collaboration with district health officials to include a mix of settings (urban, rural, conventional, and Quranic) in each district.

### Sample size

Based on a previous study in 2019 on asymptomatic malaria in Senegal (Badiane et al., 2021) reporting a prevalence of ~40%, the required sample size was calculated using a standard formula for cross-sectional surveys (Naing et al., 2022). Using a 95% confidence level (Z = 1.96), an expected prevalence *p* = 0.40, and a margin of error *i* = 0.05, the minimum sample size per district was estimated to be 738 children. To account for potential non-response or dropouts and to ensure robust statistical power for subgroup analyses, approximately 1,000 children were targeted per district. In Diourbel district, both Quranic and conventional schools were included to capture potential differences in transmission.

### Data collection method

Survey data were collected via a structured questionnaire administered on electronic tablets by trained interviewers. (In instances of technical difficulties, paper forms were used and later digitized.) The questionnaire gathered information on each child’s socio-demographic characteristics (age, sex, and residence), school type (conventional vs. Quranic), recent travel history (to capture mobility), use of malaria prevention measures (e.g., sleeping under insecticide-treated nets), and recent participation in SMC programs. RDT results were recorded for each child. The geographic coordinates (latitude, longitude) of each school were recorded using the tablet’s GPS to map survey locations.

All collected data were uploaded to a secure server and stored in a Microsoft Access relational database. Data quality was ensured through daily checks and validation rules embedded in the electronic forms (e.g., logical ranges for age, internal consistency checks).

### Laboratory methods

#### Rapid diagnostic test

After obtaining informed consent, capillary blood samples were collected by finger prick from each participating child. One drop of blood (~5 µL) was immediately used to perform a malaria RDT (SD Bioline Malaria Ag *Pf*, Standard Diagnostics, Republic of Korea), which detects *P. falciparum* histidine-rich protein 2 (HRP2). The RDT was administered and read according to the manufacturer’s instructions by trained study staff. In addition, for laboratory analyses, three to four larger drops of blood (approximately 50–100 µL in total) were spotted onto Whatman 903 filter paper (Sigma-Aldrich, St. Louis, MO, USA) for each child, creating dried blood spots (DBS). The DBS were air-dried and then stored individually in sealed plastic bags with desiccant (silica gel) at ambient temperature until further processing for molecular and serological tests.

#### Bead-based immunoassay

High-throughput bead-based immunoassay was used to measure HRP2 antigen levels in the dried blood spot samples. The assay followed the standard protocol described by Rogier et al. (2019) for multiplex antigen detection. In brief, antigen-coupled fluorescent beads (Luminex MagPlex microspheres) were added to a 96-well plate along with sample lysate from the DBS. After incubation, a biotinylated detection antibody specific to *P. falciparum* HRP2 was added. (In our assay platform, a secondary reagent that binds human IgG/IgG4 was not used, as it was focused on antigen detection rather than antibody detection.) Then, streptavidin conjugated to phycoerythrin (PE) was added to bind the biotinylated detection antibodies. Following additional incubation and wash steps, the plate was read on a MAGPIX^®^ instrument (Luminex Corp., Austin, TX, USA), which detects the median fluorescence intensity (MFI) from at least 50 beads per antigen per sample. Each sample was run in duplicate; the background-corrected MFI for HRP2 (sample MFI minus the MFI of negative-control wells containing only buffer).

To distinguish positive from negative HRP2 results in the continuous assay, a quantitative cutoff was determined. A finite mixture model was applied to the distribution of log10-transformed HRP2 concentrations. This statistical approach fits a two-component mixture (representing a negative/low group and a positive group) using the Expectation-Maximization (EM) algorithm and maximum likelihood estimation, as implemented in the mixtools package (version 2.0.0), specifically the normalmixEM() function in R version 4.3.2. Samples with HRP2 concentrations above this threshold were classified as HRP2 antigen-positive, whereas those below were considered HRP2-negative (or very low antigen levels).

HRP2 concentrations were quantified from MFI values using a validated four-parameter logistic (4PL) regression model fitted to a calibration curve of recombinant HRP2. The formula used for conversion (Plucinski et al., 2017b) was:


$$Concentration\left(ng/mL\right)=2784.84\times {\left[{\left(\frac{17316.4+13367}{MFI+7.13367}\right)}^{1.9658}-1\right]}^{-0.560}$$

which was derived from a standard dilution series of HRP2​. (This equation was used to estimate the HRP2 concentration corresponding to any given sample MFI.)

#### Molecular confirmation of infection

To confirm RDT results and resolve discrepancies between positive RDT and bead assay findings, molecular testing was performed on the dried blood spots. We employed a multiplex Photo-Induced Electron Transfer PCR (PET-PCR) assay targeting the *Plasmodium falciparum* 18S rRNA gene, as previously described by Lucchi et al. (2013). Briefly, genomic DNA was extracted from a punch of each DBS using a Qiagen blood DNA kit. The PET-PCR reactions were carried out in 20 μL volumes containing species-specific forward and reverse primers and a pair of fluorogenic “light-up” probes that become fluorescent upon binding to their PCR product. The cycling conditions were 95°C for 10 minutes, followed by 45 cycles of 95°C for 15 seconds and 60°C for 1 minute. Amplification was monitored in real-time, and a cycle threshold (Ct) value <35 was interpreted as positive for *P. falciparum* DNA. Each run included a positive control (genomic DNA from cultured *P. falciparum* 3D7 strain) and two negatives: a no-template water control and a negative human blood control (malaria-free donor). This PET-PCR method can detect low-level parasitemia and was used primarily to double-check samples where RDT and HRP2 bead assay results were discordant (RDT-positive but HRP2-negative). An RDT false-negative was defined as a case with a negative RDT result but a positive HRP2 bead assay result. Conversely, an RDT false-positive was defined as a case with a positive RDT result but a negative HRP2 bead assay result; any such RDT-positive/HRP2-negative cases were further checked by PCR to determine if they were true parasite-positive cases (which would suggest the bead assay missed the antigen, perhaps due to *pfhrp2* deletion or very low levels) or if they were likely RDT false-positives.

### Statistical analysis

To evaluate the agreement between HRP2 positivity as defined by the Gaussian mixture model and RDT results, a classification rule was applied using the model-derived cutoff. Samples with a logHRP2 value greater than or equal to the cutoff were classified as HRP2-positive; those below the threshold were classified as HRP2-negative. A confusion matrix was constructed comparing these HRP2-based classifications to RDT outcomes.

A non-parametric LOESS curve was generated for each district to depict the relationship between the probability of obtaining a positive RDT result and the log HRP2 concentration. Specifically, the HRP2 concentration corresponding to 50%, 75%, 90%, and 95% probability of a positive RDT (LOD_50_, LOD_75_, LOD_90_, LOD_95_, respectively) was derived and computed 95% confidence intervals for these estimates.

A multivariable logistic regression was also performed to assess factors influencing RDT positivity. Explanatory variables included log10 HRP2 concentration (continuous), age group (>10 years vs. ≤10 years), and sex, with district as a stratifying variable. This analysis yielded adjusted odds ratios for RDT positivity associated with HRP2 level and with older age, adjusting for other factors.

The goodness-of-fit of the logistic regression models assessing the relationship between log-transformed HRP2 concentration and RDT positivity was evaluated using the Hosmer-Lemeshow (HL) test. This test assesses the agreement between observed outcomes and predicted probabilities across deciles of risk. It was applied to both the univariate model (RDT ~ logHRP2) and the multivariate model including age and sex as covariates. The HL test was conducted using the hoslem.test() function from the ResourceSelection R package (version 0.3-6). A non-significant p-value (p > 0.05) indicated acceptable model calibration.

All analyses were conducted in R version 4.3.0 (2023-04-21).

### Ethical considerations

The study protocol was reviewed and approved by the Ethics Committee of the Senegalese Ministry of Health and Social Action (Protocol SEN14/49), as well as the Institutional Review Board at the Harvard T.H. Chan School of Public Health (IRB #CR-16330-07). Meetings were held with community leaders, local health workers, and school officials in each district to discuss the project’s objectives and procedures. Community consent was obtained from village and religious leaders where appropriate. Written informed consent was obtained from the parent or guardian of each participating child, and assent was obtained from older children (typically those 12 years and above) as per ethics guidelines. Participation was completely voluntary, and children (or their guardians) could opt out at any time. All data were kept confidential, and test results were communicated to the child’s parent/guardian along with referral to the nearest health facility for any RDT-positive cases, in line with national treatment guidelines.

## Results

### Characteristics of the study population

A total of 3,748 children were enrolled across the three districts (Diourbel: 1,906; Kedougou: 990; Tambacounda: 852). The median age of participants was 9 years (range 6–16 years), and 43.2% of the children were female. All enrolled children were generally in good health on the day of screening (by inclusion criteria, visibly ill children were referred for care and not included in the survey). **Table 1** summarizes the demographic characteristics and RDT results by district and school. The age distribution and sex ratio were similar in each district’s sample, except that the Quranic schools in Diourbel consisted solely of boys.

**Table 1:** Summary of participant characteristics and RDT prevalence by district and school.

District	School/dara	Total	Median age (range)	% Female	RDT positive (n)	Positivity rate (%)
Diourbel (n = 1906)	School-Cite Basse	488	9 (6-15)	52.3	11	2.3
	School-Sessene	495	10 (6-16)	54.1	5	1.0
	Dara-Cheikh Gueye	536	11 (6-14)	0	29	5.4
	Dara-Cheikh lbrahima Diankha	253	11 (6-16)	0	9	3.6
	Dara-Serigne Baye Lamine Ndong	134	11 (6-15)	0	3	2.2
Kedougou (n = 990)	School-Bandafassi	192	9 (6-15)	55.7	8	4.2
	School-Lamigna	196	9 (6-14)	58.2	53	27.0
	School-Samekouta	410	9 (6-15)	49.5	92	22.4
	School-Thiabedji	192	9 (6-14)	57.3	16	8.3
Tambacounda (n = 852)	School-Ndemo gayo	185	8 (6-13)	51.4	7	3.8
	School-Ndoga Babacar	313	10 (6-15)	55.9	9	2.9
	School-Samba khoreydia Wolof	104	9 (7-13)	54.8	5	4.8
	School-Seoro	137	10 (6-14)	55.5	13	9.5
	School-Sinthiou kolinka	113	9 (6-13)	58.4	10	8.8
Overall		3748	9 (6-16)	43.2	270	7.2

Overall, 7.2% (270/3,748, 95% CI: 6.4–8.0%) of participants tested positive by RDT for *P. falciparum* antigen. This overall prevalence, however, masked significant heterogeneity by region and even by school (**Table 1**).

In Diourbel (five schools surveyed, *n* = 1,906), RDT positivity was low across all sites (overall 3.0%, 95% CI: 2.4–3.7%). The point prevalence ranged from 1.0% (5/495, 95% CI: 0.1–1.9%) at School-Sessène to 5.4% (29/536, 95% CI: 3.5–7.2%) at Dara Cheikh Gueye (a Quranic boarding school), which was the only site in Diourbel with an RDT positivity above 3%. The three Quranic schools in Diourbel (Dara Cheikh Gueye, Dara Cheikh Ibrahima Diankha, and Dara Serigne Baye Lamine Ndong) had slightly higher combined positivity (approximately 4.3%, 95% CI: 3.2–5.3%) compared to the two conventional schools (2.0%, 95% CI: 1.1–2.9%).

In Kédougou (four schools, *n* = 990), RDT positivity rates were higher (overall 15.9%, 95% CI: 13.5–18.4%) than in Diourbel. The lowest prevalence in Kédougou was observed at School-Bandafassi (4.2%, 8/192, 95% CI: 1.3–7.1%), while the highest was at School-Lamigna (27.0%, 53/196, 95% CI: 21.1–33.0%). Two other schools, Samekouta and Thiabédji, had prevalences of 22.4% (95% CI: 17.6–27.2%) and 8.3% (95% CI: 4.4–12.1%), respectively.

In Tambacounda (five schools, *n* = 852), RDT positivity varied between sites, with an overall prevalence of 7.6% (95% CI: 5.8–9.5%). The lowest rate was at School-Ndoga Babacar (2.9%, 9/313, 95% CI: 1.0–4.8%) and the highest at School-Seoro (9.5%, 13/137, 95% CI: 4.5–14.5%). Other schools ranged from 3.8% (95% CI: 1.3–6.3%) to 8.8% (95% CI: 4.4–13.2%). Tambacounda’s school-to-school variation was less extreme than in Kédougou.

### Model calibration and fit

The performance of the logistic regression models was evaluated using the Hosmer-Lemeshow goodness-of-fit test. For the univariate model, which included only log-transformed HRP2 concentration as a predictor, the test yielded a chi-squared statistic of 6.42 with 8 degrees of freedom (p = 0.601), indicating good model fit. The multivariate model, which additionally included age and sex, also demonstrated adequate fit with a chi-squared statistic of 9.45 (df = 8, p = 0.306). These results support the appropriateness of the logistic models used to characterize the probability of RDT positivity across varying HRP2 concentrations. Hosmer-Lemeshow test results for both models are provided in **Supplementary Table S3**.

### Performance of RDT versus HRP2 antigen detection

Using the mixture model approach described in the Methods, a cutoff of log10 HRP2 = 0.59 was identified to distinguish HRP2-positive from HRP2-negative individuals in our bead assay data. This threshold (**Supplementary Figure S1**) separated the lower-density (presumably uninfected or very low parasitemia) distribution from the higher-density distribution of HRP2 readings.

Using HRP2 positivity (as defined by the mixture model) as the reference standard, the performance of RDT results was evaluated. Based on the confusion matrix, the RDT yielded overall a sensitivity of 31.9%, and a specificity of 99.1% (**Table 2**). The full matrix detailing for each district is shown in **Supplementary Table S1**.

**Table 2:** Overall agreement between HRP2 classification (Mixture Model) and RDT results.

RDT status	HRP2 Antigen Negative	HRP2 Antigen Positive	Total
RDT Negative	2,628	447	3,075
RDT Positive	23	210	233
Total	2,651	657	3,308

Accuracy: 85.8%.

Sensitivity: 32.0%.

Specificity: 99.1%.

To investigate discrepancies between RDT and HRP2 antigen detection, cases where RDT and HRP2 results diverged were examined. In School-Lamigna (Kédougou), for instance, 30 children were RDT-positive but had HRP2 concentrations near or below the threshold (potential false-positive RDTs). Of these, PCR confirmed *P. falciparum* infection in 21 (true positives that the bead assay may have misclassified or just below the cutoff), whereas 9 had no detectable parasite DNA. In School-Samekouta, 23 children were RDT-positive with low HRP2; PCR confirmed infection in 17 of them. In School-Seoro (Tambacounda), three such cases occurred, and one was confirmed by PCR. In Diourbel’s Dara Cheikh Gueye, six children were RDT-positive with low HRP2, and PCR confirmed infection in 5 of those. These PCR results suggest that most RDT-positive/low-HRP2 cases were true infections.

**Figure 2:**
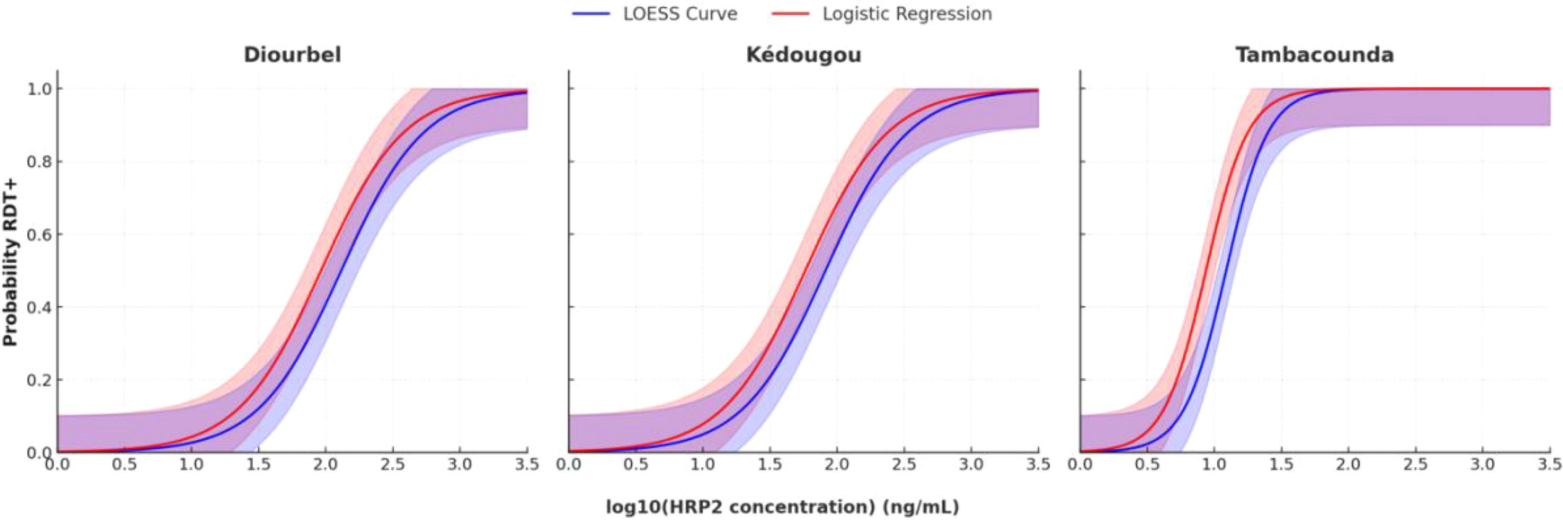
Probability of RDT positivity in relation to HRP2 concentration across districts. The LOESS curves (blue line) and logistic regression models (red line) show the probability of RDT positivity as a function of log10 HRP2 concentration. Shaded areas represent the 95% confidence intervals of the LOESS and logistic models.

For each district, the probability of a positive RDT result as a function of HRP2 concentration. LOESS curves (non-parametric) and logistic regression curves largely agree at each site (**Figure 2**). In Tambacounda, the logistic model estimated an HRP2 concentration of 1.3 ng/mL for 50% RDT positivity (LOD_50_) and 3.9 ng/mL for 95% positivity (LOD_95_). In contrast, Diourbel, a low-transmission region, showed much higher thresholds, with an LOD_50_ of 9.5 ng/mL and LOD_95_ of 204.3 ng/mL. Kédougou exhibited intermediate values, with an LOD_50_ of 6.0 ng/mL and LOD_95_ of 121.2 ng/mL. The corresponding estimated HRP2 detection thresholds (LOD_50_ to LOD_95_) in each district are provided in **Supplementary Table S2**.

Multivariate analysis confirmed that HRP2 concentration remained the strongest independent predictor of RDT positivity (adjusted OR: 14.55, 95% CI: 11.14–19.00, p < 0.001). After adjusting for HRP2 levels and district, age group was not statistically significant, although children older than 10 years showed a non-significant trend toward reduced odds of being RDT-positive compared to those 10 years or younger (adjusted OR: 0.76, 95% CI: 0.51–1.13, p = 0.171). District was significantly associated with RDT positivity: children from Tambacounda and Kédougou had higher odds of testing positive compared to those from Diourbel, with adjusted ORs of 7.42 (95% CI: 3.76–14.64) and 3.02 (95% CI: 1.79–5.08), respectively (**Table 3**).

**Table 3:** Factors influencing sensitivity of HRP2-based RDT performance as assessed using multivariate logistic regression across study districts.

Factors	Adjusted odds ratio	95% CI	*p-value*
Intercept	0.00	0.0–0.01	
Age > 10 years	0.76	0.51–1.13	0.171
Kédougou	3.02	1.79–5.08	< 0.001
Tambacounda	7.42	3.76–14.64	< 0.001
log_10_(HRP2 concentration)	14.55	11.14–19.00	< 0.001

Reference categories are age ≤10 years and Diourbel district. Gender was excluded from the final model due to non-significant contribution.

## Discussion

RDTs are quick and field-deployable, making them indispensable for routine diagnosis and NMCP-led surveys. However, their limit of detection (roughly 50–200 parasites/μL of blood) means that some subclinical infections go undetected (Diallo et al., 2017; Oviedo et al., 2023). In this study a strong concordance between RDT results and the highly sensitive bead-based HRP2 antigen assay was observed across most survey sites. The findings in this study are consistent with data from a Nigerian household survey, which showed that *P. falciparum* prevalence measured by conventional RDTs closely matched prevalence measured by an HRP2 bead-based assay (Oviedo et al., 2023). This suggests that standard HRP2-based RDTs, although they have lower analytical sensitivity than PCR or lab immunoassays, capture the majority of patent infections in moderate-to-high transmission settings. The relatively low sensitivity (32.0%) and high specificity (99.1%) of RDTs when compared to the mixture model–based HRP2 classification highlight the differing sensitivities of these diagnostic tools. The bead-based assay is capable of detecting HRP2 at concentrations well below the detection threshold of conventional RDTs. Consequently, when used as a reference standard, it classifies a broader set of individuals as HRP2-positive, including many with low antigen levels that are not captured by RDTs. However, it is important to note that low concentrations of circulating HRP2 do not always indicate active infection. HRP2 is known to persist in the bloodstream for several weeks after parasite clearance, especially following treatment. Therefore, some of the cases classified as false negatives by RDT—relative to the bead assay—may instead represent individuals with residual antigenemia, not true parasitemia. This distinction is particularly relevant in settings with widespread use of antimalarial interventions like SMC, where subclinical or recently treated infections may contribute to background HRP2 levels. While this limits the sensitivity estimate of RDTs, it also illustrates their practical specificity for detecting infections with sufficient antigen burden likely to correspond with ongoing parasitemia. Additionally, the possibility of *pfhrp2* gene deletions (parasites lacking the HRP2 antigen) remains a concern for any HRP2-based test (Plucinski et al., 2017b), although to date no large-scale HRP2 deletions have been documented in Senegal (Deme et al., 2014).

The results in this study highlight significant variability in the LODs of RDTs across the different epidemiological settings. In Tambacounda, the estimated LOD_95_ of 3.9 ng/mL is consistent with previously reported values from community surveys in Mozambique, where LOD_95_ thresholds as low as 2.5–3.2 ng/mL were observed (Plucinski et al., 2017a). In contrast, Diourbel, a lower-transmission region, exhibited a considerably higher LOD_95_ of 204.3 ng/mL, suggesting that only infections with high antigenemia are reliably detected, while infections with lower HRP2 concentrations may go undetected. These district-level differences are not unusual and align with reports of up to threefold variability in RDT detection thresholds even within a single country, as observed in Angola (Plucinski et al., 2017a). The findings highlight the value of complementary high-sensitivity assays in low-transmission or pre-elimination settings. Moreover, it is known that host immunity influences parasite clearance rates and circulating antigen levels; individuals in historically high-transmission areas like Kédougou and Tambacounda tend to develop stronger immunity, which can lead to lower parasite densities in the blood (often asymptomatic infections)​​. This can directly impact the sensitivity of antigen-based diagnostics like RDTs and even the bead assay.

Overall, these findings reinforce prior observations that RDTs maintain a high sensitivity in moderate-to-high transmission African settings, even when compared against more sensitive methods. For instance, in a low-transmission area of Senegal, Diallo et al. (2017) found RDTs had good sensitivity relative to PCR. A recent health facility-based study in Dakar, Senegal reported that HRP2 RDTs had high sensitivity and specificity when benchmarked against PCR and laboratory antigen detection (Badiane et al., 2022), aligning with what was observed in the school-based context. Similarly, analyses of Nigeria’s 2018 DHS survey showed a very high concordance between HRP2 bead assay results and standard RDT outcomes (Oviedo et al., 2023). A *post hoc* study testing samples from Mozambique, Angola, and Senegal with an HRP2 bead assay suggested that RDTs likely detect infections at even lower HRP2 concentrations than previously thought, implying better field sensitivity than lab estimates (Plucinski et al., 2017a). Likewise, Rogier et al. (2022) demonstrated that HRP2-specific RDTs reliably detected low antigen levels in community surveys in Tanzania, supporting the idea that these RDTs can catch low-density infections in practice. Taken together, these evidences support the observation that RDTs can effectively identify low-level infections across various transmission settings, validating their continued use in population-based malaria surveys.

However, it is important to note that in very low-endemic settings the picture may differ. In Haiti, for example, an ultrasensitive bead assay found many HRP2-positive individuals whom conventional RDTs did not detect—only about 14.5% of bead assay positives were RDT-positive, and conversely, 62.5% of RDT-positive individuals had no detectable HRP2 by the assay (Rogier et al., 2020). This discrepancy suggests that a multitude of factors can affect RDT performance under real-world conditions. This finding underscores that multiple factors—including parasite genetic factors (e.g., *pfhrp2* deletions), RDT manufacturing quality, storage conditions, and user training—can influence RDT performance in field settings (Plucinski et al., 2017a). In very low transmission or elimination settings, these factors become more critical, and more sensitive diagnostics (or a combination of diagnostics) may be needed to accurately track malaria infections.

This school-based survey also revealed marked geographic variation in malaria prevalence and antigen levels within Senegal, a pattern consistent with historical data (Programme National de Lutte contre le Paludisme (PNLP), 2019). In Diourbel, for instance, the substantially higher malaria prevalence observed in one of the Quranic boarding schools (Dara) compared to nearby conventional schools could be linked to increased exposure to mosquito bites and different living conditions. Children in residential daaras often spend more time outdoors in the evenings and nights, which heightens their risk of mosquito contact. Additionally, socioeconomic and living conditions in some daaras (e.g., crowded sleeping quarters, limited access to bed nets or healthcare) may contribute to higher vulnerability. This finding highlights the importance of targeted programs like PECA-DARA (Prise en Charge des cas à domicile pour les Dara), which provides malaria case management and preventive measures in Quranic schools. In Diourbel, a local initiative under the President’s Malaria Initiative has distributed large bed nets designed for groups of children who sleep in shared accommodations at daaras (U.S. President’s Malaria Initiative (PMI), 2023). These findings support the need for tailored interventions in such high-risk subpopulations, providing evidence for the effectiveness of focused malaria prevention strategies in these groups (Gaye et al., 2020).

### Study limitations

This study has several limitations. First, it was conducted exclusively in areas where SMC is implemented during the high transmission season. This may limit the generalizability of the study findings to areas without SMC, as the intervention could suppress parasite densities and thus influence RDT and HRP2 results (though our data collection occurred just before the next SMC round, minimizing residual drug effects). Second, microscopy was not performed in the field, and actual parasite densities were not measured by PCR, which limits the ability to directly correlate HRP2 concentrations with parasite count. Incorporating quantitative PCR or microscopy in future studies could help clarify the relationship between antigen levels and parasitemia. Third, although *pfhrp2/3* gene deletions are an emerging threat to HRP2-based diagnostics, testing for these deletions was not conducted in this survey due to resource limitations. Given increasing reports of *pfhrp2* deletions across Africa, future analyses should include surveillance for these genetic variants, particularly in cases of unexpected discrepancies between RDT and antigen or PCR results. Fourth, HRP2 concentration estimates were based on a proxy calibration curve and assumed consistent antigen recovery from dried blood spots. While a published conversion equation was used, systematic errors could influence the absolute LOD values reported, though relative comparisons across districts would remain valid. Additionally, while the two-Gaussian mixture model applied in this study provided a data-driven threshold for classifying HRP2 positivity, its performance, particularly the observed trade-off between high specificity and lower sensitivity, suggests room for refinement. More flexible mixture models, including those incorporating skewed distributions or heteroscedastic variances, may better capture the underlying distribution of HRP2 levels, especially in heterogeneous populations. Such approaches have been successfully implemented and discussed in the statistical literature (Domingues et al., 2024), and could improve the accuracy of latent class classification in similar settings. Although our choice of a two-component Gaussian model prioritized interpretability and simplicity, future applications could benefit from evaluating more complex models to enhance classification performance in antigen-based surveillance systems. Finally, some of the observed inter-district differences (e.g., lower RDT positivity in Diourbel) may be attributable to unmeasured factors such as variability in SMC adherence, vector densities, or localized transmission dynamics not captured during the survey.

## Conclusion

Integrating RDTs with high-throughput bead-based antigen detection in school surveys provides a powerful and scalable approach for malaria surveillance, especially for detecting infections that might be missed by RDTs alone. This combined strategy enables national malaria control programs to gain deeper insights into transmission patterns in the community, allowing for more targeted and timely interventions in areas of persistent transmission. The findings indicate that merging school-based surveys with advanced immunoassay tools can create a comprehensive surveillance framework that leverages the strengths of each method. In our context, standard HRP2-based RDTs proved to capture most infections, validating their use, while the bead assay provided quantitative data on infection intensity and helped identify the lower-density infections that approach the RDT detection threshold. Going forward, such integrated surveillance can guide the tailoring of interventions (like focal drug administration or vector control) to specific schools or communities, thereby accelerating progress toward malaria elimination in Senegal and similar settings.

## Data Availability

The original contributions presented in the study are included in the article/**Supplementary Material**. Further inquiries can be directed to the corresponding author.
