# Supplementary material for: RDT performance through high-throughput bead-based antigen detection during malaria school survey in Senegal
Source: Front Parasitol. 2025 May 29;4:1598280. doi: 10.3389/fpara.2025.1598280 (PMC12159022; doi:10.3389/fpara.2025.1598280)
Supplement: Supplementary file 2 [file SupplementaryFile1.docx]

**RDT performance through high-throughput bead-based antigen detection during malaria school survey in Senegal**

**Supplementary Figure**

**
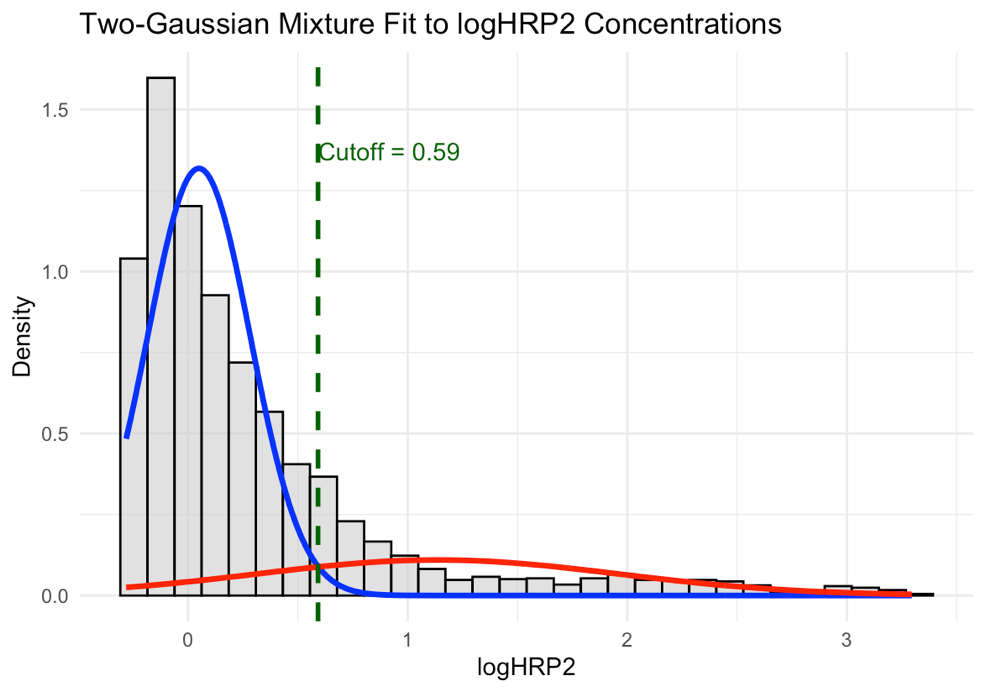
**

| **Component** | **Mean (μ)** | **Standard Deviation (σ)** | **Proportion (π)** |
| --- | --- | --- | --- |
| HRP2-negative | 0.053 | 0.221 | 75.9% |
| HRP2-positive | 1.110 | 0.816 | 24.1% |

**Figure S1.** Histogram of log₁₀-transformed HRP2 concentrations overlaid with fitted Gaussian mixture model components.

**Supplementary Tables**

**Table S1.** Agreement between HRP2 classification (Mixture Model) and RDT results

**S1-A: Diourbel**

|  | **HRP2 Negative** | **HRP2 Positive** | **Total** |
| --- | --- | --- | --- |
| **RDT Negative** | 722 | 50 | 772 |
| **RDT Positive** | 3 | 34 | 37 |
| **Total** | 725 | 84 | 809 |

- **Accuracy**: 93.4%
- **Sensitivity**: 40.5%
- **Specificity**: 99.6%

**S1-B: Kédougou**

|  | **HRP2 Negative** | **HRP2 Positive** | **Total** |
| --- | --- | --- | --- |
| **RDT Negative** | 516 | 283 | 799 |
| **RDT Positive** | 10 | 156 | 166 |
| **Total** | 526 | 439 | 965 |

- **Accuracy**: 69.6%
- **Sensitivity**: 35.5%
- **Specificity**: 98.1%

**S1-C: Tambacounda**

|  | **HRP2 Negative** | **HRP2 Positive** | **Total** |
| --- | --- | --- | --- |
| **RDT Negative** | 654 | 10 | 664 |
| **RDT Positive** | 10 | 20 | 30 |
| **Total** | 664 | 30 | 694 |

- **Accuracy**: 97.1%
- **Sensitivity**: 66.7%
- **Specificity**: 98.5%

### ****Table S2.**** Estimated RDT detection thresholds based on HRP2 concentration

### ****S2-A:**** LODs (log₁₀ MFI)

| **District** | **LOD50 (95% CI)** | **LOD75 (95% CI)** | **LOD90 (95% CI)** | **LOD95 (95% CI)** |
| --- | --- | --- | --- | --- |
| **Diourbel** | 1.965 (1.652 – 2.267) | 2.428 (2.113 – 2.814) | 2.890 (2.463 – 3.347) | 3.204 (2.701 – 3.722) |
| **Kédougou** | 1.763 (1.637 – 1.899) | 2.224 (2.047 – 2.400) | 2.684 (2.445 – 2.888) | 2.998 (2.750 – 3.262) |
| **Tambacounda** | 0.938 (0.744 – 1.164) | 1.173 (0.937 – 1.435) | 1.407 (1.102 – 1.728) | 1.566 (1.207 – 1.909) |

### ****S2-B:**** Converted LODs in ng/mL using the calibration formula

| **District** | **LOD50 (95% CI)** | **LOD75 (95% CI)** | **LOD90 (95% CI)** | **LOD95 (95% CI)** |
| --- | --- | --- | --- | --- |
| **Diourbel** | 9.5 (4.7–19.6) | 29.1 (13.5–76.2) | 92.3 (31.8–294.5) | 204.3 (57.4–797.0) |
| **Kédougou** | 6.0 (4.5–8.1) | 17.7 (11.5–27.2) | 55.0 (30.4–91.8) | 121.2 (64.9–236.9) |
| **Tambacounda** | 1.3 (1.0–1.8) | 1.8 (1.3–2.9) | 2.8 (1.6–5.5) | 3.9 (1.9–8.3) |

**Table S3.** Hosmer-Lemeshow Goodness-of-Fit Test Results

| **Model** | **Chi-Square (χ²)** | **Degrees of Freedom (df)** | **p-Value** |
| --- | --- | --- | --- |
| Univariate Logistic | 6.42 | 8 | 0.601 |
| Multivariate Logistic | 9.45 | 8 | 0.306 |
